# Midesophageal Metastatic Disease After Treatment of Gastroesophageal Junction Adenocarcinoma

**DOI:** 10.14309/crj.0000000000000869

**Published:** 2022-10-06

**Authors:** Tim Brotherton, Sam Burton, Eric Knoche, Michael Presti

**Affiliations:** 1 Department of Internal Medicine, Saint Louis University Hospital, St. Louis, MO; 2 Division of Gastroenterology and Hepatology, Saint Louis University Hospital, St. Louis, MO; 3 Division of Oncology, Washington University, St. Louis, MO; 4 St. Louis Veteran's Administration, St. Louis, MO

## Abstract

Esophageal cancer carries a significant risk of morbidity and mortality because of its poor prognosis. Adenocarcinoma is the most common type of esophageal cancer in the United States. Local metastasis within the esophagus is extremely rare and likely because of the complex esophageal lymphatic system. Our patient is a 74-year-old man with adenocarcinoma of the gastroesophageal junction treated with chemotherapy and radiation who was subsequently found to have local metastasis to the proximal esophagus.

## INTRODUCTION

Esophageal cancer is estimated to account for 1.1% of newly diagnosed cancer and 2.7% of cancer deaths annually.^1^ Owing to a poor prognosis and rising incidence, esophageal cancer causes a significant amount of morbidity and mortality.^2^ There are 2 distinct histologic types of esophageal cancer: adenocarcinoma and squamous cell carcinoma. While squamous cell carcinoma is the most common type worldwide, adenocarcinoma is more common in the United States, Australia, the United Kingdom, and Western Europe.^3^

Adenocarcinoma typically occurs in the distal esophagus, frequently arising from underlying Barrett's esophagus. Common locations of metastases for esophageal cancer include the periceliac and perihepatic lymph nodes, liver, lungs, bone, and adrenal glands.^4,5^ Local metastasis to the proximal esophagus is extremely rare. We present a case of a 75-year-old man with adenocarcinoma of the gastroesophageal junction (GEJ) treated with chemotherapy and radiation who was subsequently found to have local metastasis to the midesophagus.

## CASE REPORT

**Figure 1.**
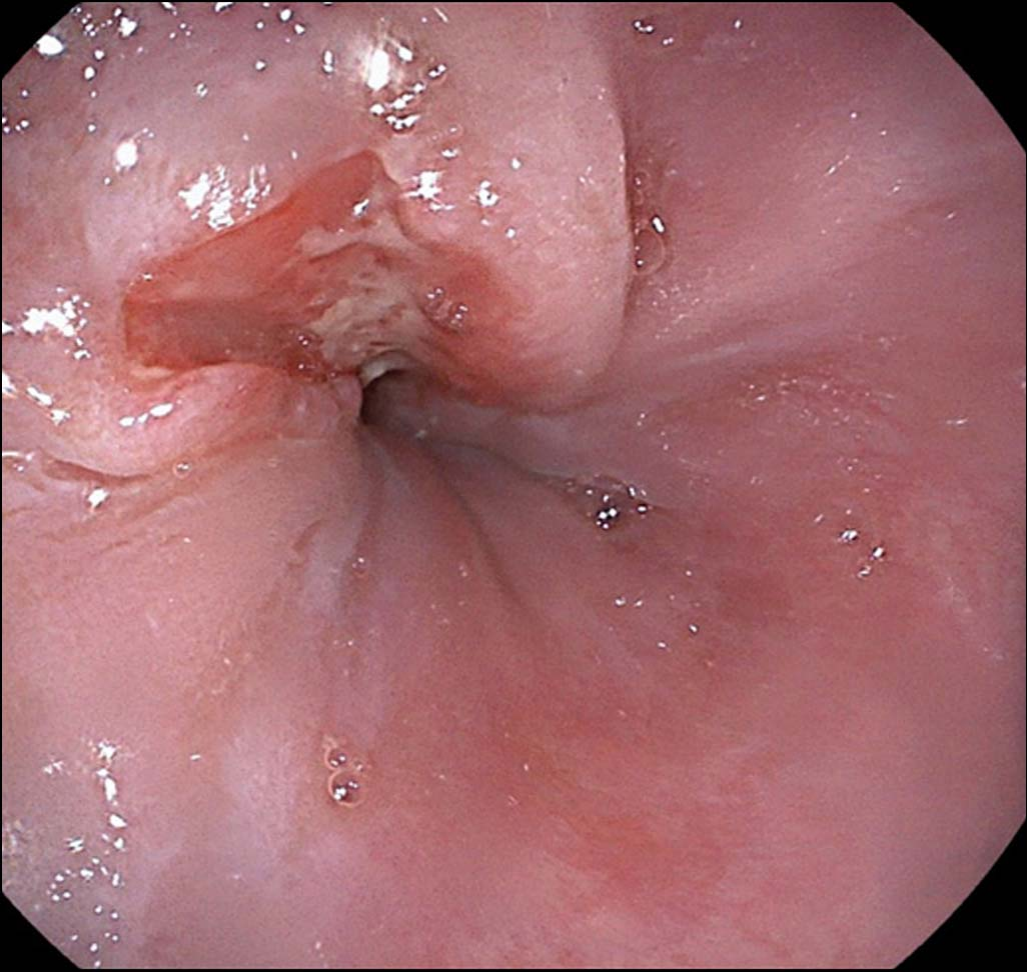
Endoscopic view of the lower esophagus, just above GEJ with ulcerated lesion. GEJ, gastroesophageal junction.

The patient is a 74-year-old man with a medical history of gastroesophageal reflux disease (GERD), chronic inactive hepatitis B infection, and mild Alzheimer dementia. He was referred to the gastroenterology clinic for evaluation of a 6-week history of progressive dysphagia to solids and a 10-pound weight loss. An esophagogastroduodenoscopy was performed, which showed a focal ulceration in the distal esophagus extending 2 cm above and 1 cm below the GEJ (Figure 1). Biopsies confirmed adenocarcinoma with immunohistochemical testing strongly positive for pancytokeratin, CK7, CK20, and CDx2, consistent with a gastrointestinal origin. Endoscopic ultrasound showed a malignant esophageal tumor at the GEJ that was staged as T3N0Mx without any signs of lymphatic metastasis. Positron emission tomography-computed tomography (PET-CT) showed moderate fluorodeoxyglucose increased activity in the GEJ/gastric cardia with associated mucosal thickening on CT. Findings were consistent with biopsy-proven poorly differentiated GEJ carcinoma. He was not a surgical candidate and subsequently underwent treatment with chemotherapy and radiation with a curative intent. He required no dose adjustment nor interruption of chemotherapy or radiation. Symptomatically, he improved, with resolution of dysphagia, and started to regain weight.

**Figure 2.**
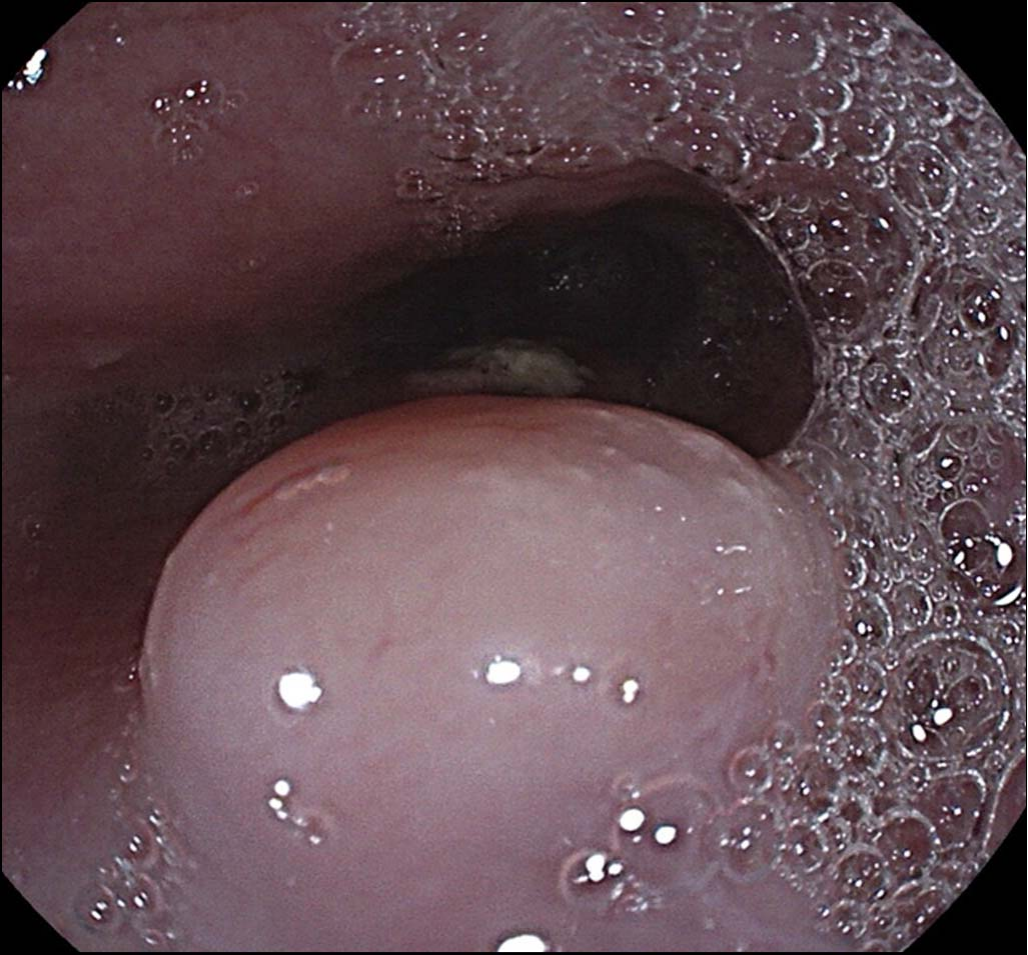
Endoscopic view of the proximal esophagus with raised bulbous lesion.

**Figure 3.**
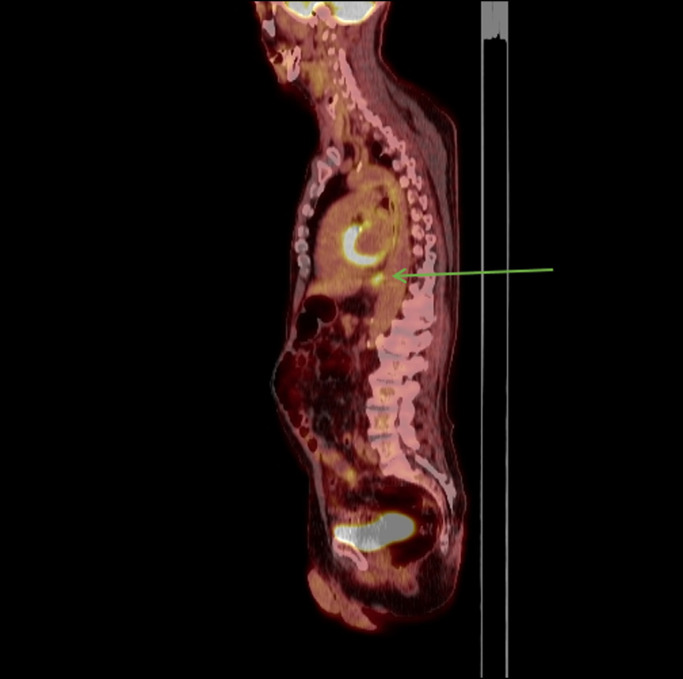
Initial PET scan with hypermetabolic activity at GEJ consistent with adenocarcinoma.

**Figure 4.**
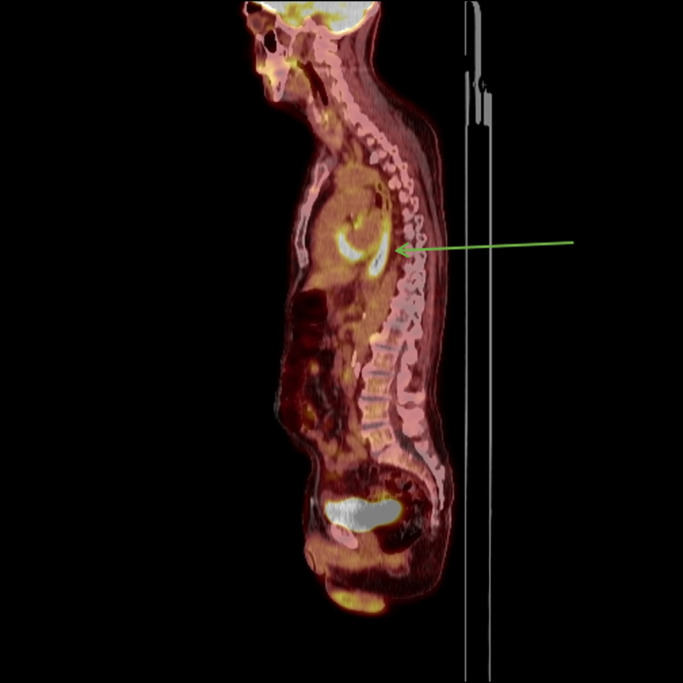
Follow-up PET scan with increased metabolic activity at GEJ and extending up to the proximal esophagus (arrow).

Approximately 3 months later, he began to have worsening dysphagia, odynophagia, and weight loss. A repeat esophagogastroduodenoscopy was performed. The ulcerated GEJ lesion appeared larger in size. Several focal raised lesions were seen proximally in the midesophagus (Figure 2). With clear areas of normal mucosa between the ulcerated cancer and these new discrete raised lesions, these findings were initially believed to be secondary to radiation treatment. Biopsies were consistent with local metastatic disease in the proximal areas of the midesophagus. Repeat PET-CT showed an interval significant increase in size and metabolic activity of the previously seen mass in the GEJ and cardia of the stomach, with an interval development of hypermetabolic activity in the mid through distal esophagus. The findings were consistent with biopsy-proven progression of disease (Figures 3 and 4). The patient was subsequently initiated on palliative chemotherapy.

## DISCUSSION

Esophageal cancer is a major cause of morbidity and mortality, representing the sixth most common cause of cancer-related deaths worldwide.^2,6,7^ Primary risk factors of esophageal cancer include BE, GERD, and obesity, with cigarette smoking also representing a moderate risk factor.^7–9^

Staging of disease is the most important prognostic factor. Patients with metastatic esophageal cancer have an estimated 5-year survival rate of 5%.^2^ The 3 main modalities for the spread of esophageal cancer include direct spread through tissue, lymphatic, or hematogenous spread. The esophagus has no serosal coating allowing for tumor ingrowth and early metastasis through direct spread.^10^ The complex esophageal lymphatic system can explain invasion into neighboring anatomical structures.^11^ Lymphatic vessels exist in all 3 layers of the esophagus, and esophageal cancer has a high rate of lymph node metastasis.^12^ Up to 10% of patients with mucosal cancer and 50% of those with submucosal cancer may already have concomitant lymph node involvement at diagnosis.^13^

In our patient, contiguous tumor spread is unlikely given normal mucosa between initial malignant tumor at GEJ and new proximal malignant lesions seen on endoscopy as well as 2 areas of noncontiguous, distinct uptake on follow-up PET-CT. Although malignant lymph node involvement was not seen on the initial PET-CT, staging endoscopic ultrasound, or follow-up PET-CT after chemoradiation, spread of malignant cells to the proximal esophagus by lymphatics is plausible. Moreover, the lymphatic vessels in the lamina propria and submucosa primarily run longitudinally and are the bases for bidirectional drainage; thus, they may have facilitated proximal spread as seen in our case.^12^ The presence of a metachronous lesion or new primary cancer is unlikely because of the concordance of the pathology.

Other case reports have suggested a role of GERD in the metastasis of gastric cancer to the esophagus (ie, the reflux of cancer cells from the stomach resulting in implant metastasis).^14,15^ Another possibility is tumor seeding from the stomach into the esophagus because of prior endoscopy. While our patient had a primary GEJ tumor and not a gastric tumor, it is still reasonable for either of the above described modalities of metastasis to have occurred because his cancer disseminated from the distal to proximal midesophagus. Furthermore, this patient had a history of GERD and underwent endoscopy before metastasis.

In conclusion, we present a very unusual case of esophageal adenocarcinoma of the GEJ with local metastasis to the proximal midesophagus. The mechanism of this atypical form of metastasis is likely because of the complex esophageal lymphatic system.

## DISCLOSURES

Author contributions: T. Brotherton and S. Burton wrote the initial draft of the report. E. Knoche and M. Presti served as supervising physicians on the case who reviewed and edited the manuscript prior to submission. T. Brotherton is the article guarantor.

Financial disclosure: None to report.

Informed consent was obtained for this case report.
